# Down-regulation of metabolic proteins in hepatocellular carcinoma with portal vein thrombosis

**DOI:** 10.1186/s12014-017-9164-y

**Published:** 2017-08-02

**Authors:** Wei-Chen Lee, Hong-Shiue Chou, Ting-Jung Wu, Chen-Fang Lee, Pao-Yueh Hsu, Hsiu-Ying Hsu, Tsung-Han Wu, Kun-Ming Chan

**Affiliations:** Division of Liver and Transplantation Surgery, Department of General Surgery, Chang-Gung Memorial Hospital, Chang-Gung University College of Medicine, 5, Fu-Hsing Street, Kwei-Shan Township, Taoyuan, Taiwan

**Keywords:** Vascular invasion, Fumarate hydratase, Carbonic anhydrase, Pseudohypoxia, Hepatocellular carcinoma

## Abstract

**Background:**

Hepatocellular carcinoma is an aggressive malignancy with poor prognosis and easy to recur even the tumor is totally removed by surgery. Portal vascular invasion is one of the major factors contributing to tumor recurrence and poor prognosis. However, why hepatocellular carcinoma is easy to grow into vessels is unclear.

**Methods:**

Surgical specimens from seven hepatocellular carcinoma patients with portal vein thrombosis and seven patients without vascular invasion were utilized to analyze protein expression by proteomic technique. The proteins in the tumors were separated by 2-dimensional electrophoresis. Protein patterns in the gels were recorded as digitalized images. The differences of expression in hepatocellular carcinoma with or without portal vein thrombosis were identified by mass spectrometry.

**Results:**

Clinically, the tumors with portal vein thrombosis were larger than those without portal vein thrombosis. The median survival time for the patients with portal vein thrombosis was much shorter [4 (ranged 2.5–47) vs. 53 (ranged 33–85) months, *p* = 0.002]. By analyzing the protein expression in cancer tissues with or without portal vein thrombosis, the differences of protein expression were mainly metabolic enzymes. Carbonic anhydrase I, betaine–homocysteine *S*-methyltransferase 1, fumarate hydratase, isovaleryl-CoA dehydrogenase, short-chain specific acyl-CoA dehydrogenase and arginase-1 were all down-regulated in the tumors with portal vein thrombosis.

**Conclusion:**

Metabolic enzymes and cytosol carbonic anhydrases were downregulated in hepatocellular carcinoma with portal vein thrombus. The deficiency of metabolic enzymes and cytosol carbonic anhydrases may alter cellular metabolisms and acid–base balance in hepatocellular carcinoma, which may facilitate to invade portal vein.

## Background

Hepatocellular carcinoma (HCC), the major primary liver cancer, is the sixth most common malignancy in the world [1]. This malignancy is aggressive and the prognosis is poor if the tumors are found at a late stage [2, 3]. Even if the tumors are found at an earlier stage and removed totally by liver resection or liver transplantation, the treatment results are still not satisfactory because the tumors are easy to recur [4, 5]. In liver resection, tumor recurrent rate is as high as 65% within 3 years [4]. In liver transplantation, the tumors may recur in a small proportion of patients even if the tumors are within Milan criteria [6, 7]. If liver transplantation is performed for an advanced HCC, tumor recurrence is a predestination although native liver was removed and a new liver was implanted [8]. Undoubtedly, tumor recurrence remains a critical and unsolved issue which interferes with a successful treatment for HCC no matter liver resection or liver transplantation is performed.

Many prognostic factors are related to tumor recurrence after liver resection or liver transplantation [9, 10]. By biostatistics analysis, vascular invasion is always one of the most important risk factors of tumor recurrence either in liver resection or liver transplantation [4, 11, 12]. Why HCC facilitates to grow into portal venules is still a mystery. The only thing known is that the incidence of vascular invasion for HCC increases when the tumor size becomes large [13]. The Liver Cancer Study Group of Japan reported that the incidence of portal vein thrombosis (PVT) was 62% in autopsy [14]. Portal vein thrombosis is an extreme form of vascular invasion for HCC. Once upon PVT is noted, intrahepatic metastasis and distal metastasis are intended to occur. Portal vein thrombosis has been described as one of the three independent factors to predict shorter survival for untreated HCC [15]. In another study, PVT was also mentioned as one of the most robust predictors to predict death [16]. The life span for the patients having PVT in 1st or 2nd branches of portal veins is only 4–6 months. If PVT approaches to main portal vein, all treatments are not effective and the life span is reduced to 2–3 months only [17]. Thus, it is essential to understand why HCC invades vessels.

Compared to other malignancies, PVT is a special characteristic of HCC. It also provides an opportunity to understand why HCC invades and grows into blood vessels. In this study, surgical specimens of HCC with PVT or without microvascular invasion were collected. Proteomic technique was applied to investigate the different expression of proteins in HCC with PVT and HCC without vascular invasion.

## Methods

### Surgical specimen

The surgical specimens of HCC with macroscopic portal vein thrombosis were utilized to undergo this study. Surgical specimens from seven HCC patients with PVT were obtained from tumor tissue bank at Chang-Gung Memorial Hospital. For comparison of HCC with or without vascular invasion, another 7 surgical specimens from HCC patients without microvascular invasion were obtained, too. The utilization of surgical specimens for this study was approved by Ethic Committee of Chang-Gung Memorial Hospital.

### Tissue sample preparation

Tissue samples were prepared as described by Li et al. [18]. In brief, frozen tissue samples (200 mg) were washed with cooled PBS for 3 times and homogenized in a cooled mortar and pestle. The obtained cells were dissolved in lysis buffer (containing 8 M urea, 4% CHAPS, 65 mM DTT) and sonicated on ice for ten rounds of 10 s. The lysate, then, was centrifuged for 1 h at 12,000 rpm to remove unsolved cell debris.

### 2-Dimensional gel electrophoresis (2-DE) [19]

Isoelectric focusing (IEF) was performed using commercially available, dedicated apparatuses: Protean IEF Cell (BioRad, Hercules, CA). Immobilized pH gradient (IPG) strips were used in accordance with the manufacturer’s instructions. Samples containing 450 µg for SYPRO Ruby staining was diluted to 300–350 mL with rehydration solution (7 Murea, 2 M thiourea, 4% CHAPS, 65 mM DTT, 0.2% v/v pH 3–10 pharmalyte, trace Bromophenol blue), and applied to strips by 12 h rehydration at 50 V. Proteins was focused subsequently for 30 min at 200 V, 30 min at 500 V, 30 min at 1000 V, 30 min at 5000 V, and finally 6 h at 10,000 V to give a total of 70 kVh. All IEF was carried out at 20 °C. After the first-dimensional IEF, IPG strips were placed in an equilibration solution (6 M urea, 2% SDS, 30% glycerol, 50 mM Tris–HCl, pH 8.8) containing 1% DTT and were shaken for 10 min at 50 rpm on an orbital shaker. The strips were then transferred to the equilibration solution containing 2.5% iodoacetamide and shaken for another 10 min before being placed on a 12.5% polyacrylamide gel slab. Separation in the second dimension was carried out using Protean II electrophoresis equipment and Tris–glycine buffer (25 mM Tris, 192 mM glycine) containing 0.1% SDS, at a current setting of 5 mA/gel for the initial 1 h and 10 mA/gel thereafter. The second dimensional SDS-PAGE was developed until the bromophenol blue dye marker had reached the bottom of the gel.

### Protein visualization and image analysis

The gels were initially fixed for 30 min in a buffer containing 10% methanol and 7% acetic acid. Then, the gels were stained for 3 h in a commercially available SYPRO Ruby buffer (Molecular Probes, Eugene, OR) followed by washing in the fixation buffer. Protein patterns in the gels were recorded as digitalized images using a high-resolution scanner (GS-710 Calibrated Imaging Densitometer, BioRad, Hercules CA). The SYPRO Ruby-stained gels were used for image analysis using the Progenesis software package (Progenesis Discovery, Nonlinear Dynamics, Durham, NC).

### Protein identification by MS

Protein spots with column volume >20% and *p* < 0.001 (ANOVA) were selected for protein identification. Protein spots were excised manually from the SYPRO Ruby-stained 2-D gels. The excised spots were de-stained using 50 mM NH_4_HCO_3_ in 50% ACN and dried in a SpeedVac concentrator. Protein was then digested by incubation overnight at 37 °C with 5 ng/mL trypsin (Promega, Madison, WI) in 50 mM NH_4_HCO_3_, pH 7.8. Tryptic peptides were extracted from the gel pieces in one volume 0.1% TFA while vortexing for 5 min followed by sonication for 5 min. Crude digest mixtures were concentrated and desalted using C18 ziptips (Millipore) followed by being eluted in 1.5 mL matrix (5 mg CHCA/mL in 50% ACN/0.1% TFA) for MALDI-TOF MS and MS/MS analysis (Ultra-flex II, Bruker Daltonics). Protein ID was searched against the NCBI and Swiss-Prot database, using MASCOT software from matrix science (www.matrixscience.com).

### Western blot

Proteins from the 14 paired tumor and non-tumor tissues were separated on 12% polyacrylamide gels and transferred to polyvinylidene difluoride membranes (Amersham Biosciences, Uppsala Sweden). These blots were incubated for 1 h at room temperature in Tris-buffered-saline with Tween (20 mM Tris–Cl, 140 mM NaCl, pH 7.5, 0.05% Tween 20) containing 5% skim milk. Primary antibodies used were anti-betaine–homocysteine *S*-methyltransferase 1 (BHMT), carbonic anhydrase I (CA I), and fumarate hydratase (FH) monoclonal antibody (diluted 1:1000, Abcam, Cambridge MA). Blots were incubated with primary antibodies overnight at 4 °C. After washing three times in Tris-buffered-saline with Tween, blots were incubated with horseradish peroxidase-conjugated secondary antibody (diluted 1:2000, Santa Cruz Biotechnology) for 1 h at room temperature. Immunoreactive complexes were visualized using enhanced chemiluminescence (ECL) reagents (Millipore, Billerica, MA).

### Immunohistochemical stain

The antibodies used for immunohistochemistry were the same as those used for Western blotting analysis. Six-micron-thick sections were deparaffinized in xylene and then rehydrated through graded alcohols to distilled water. The primary antibodies against BHMT, CAI and FH were used at dilutions of 1:100. The antibody complex was detected using an UltraVision Quanto Detection System HRP DAB Kit (Thremo scientific, Waltham MA). Slides were counterstained with hematoxylin for 20 s, dehydrated in 100% ethanol and xylene, and then covered with slips mounted with DPX mountant (BDH). The primary antibody was replaced with PBS as a negative control.

### Statistical analysis

The comparisons of categorical variables were determined by χ^2^ or Fisher’s Exact Tests if the test number was less than five. The significance of the differences between the two groups was determined by Mann–Whitney U test (SigmaStat 3.0 software, Statistical Solutions Limited, Cork, Ireland). A *p* value below 0.05 for clinical presentation and protein expression were considered to be significantly different because the patient number was limited.

## Results

### Characteristics of patients

In this study, surgical specimens were taken from two groups of patients with different pathological presentation: HCC with PVT and HCC without vascular invasion. The clinical profiles and characteristics of tumors were collected and compared. The results showed that the patients with PVT were younger than the patients without vascular invasion. In their tumor characteristics, the analytic results showed that the tumors with PVT were larger than those without PVT and had a higher trend of non-encapsulation. The other characteristics including presence of daughter nodules, differentiation grading and production of alpha-fetoprotein were not different between the two groups. The survival time between the two groups of patients was markedly different. The median survival time for the patients with PVT was only 4 months although the tumors were removed completely. On the other hand, the median survival time for the patients without vascular invasion was 53 months until now. Up to date, five of seven patients were still alive without tumor recurrence (Table 1).

**Table 1:** The clinical profiles and tumor characteristics of 7 HCC patients with PVT and another 7 patients without PVT

	PVT (n = 7)	No PTV (n = 7)	*p*
Gender (M/F)	7/0	5/2	0.462
Age [median(range)]	47 (39–71)	69 (53–79)	0.016
Operation			
Lobectomy	4	0	0.070
Segmentectomy	3	7	
Tumor			
Size [median(range)] (cm)	8 (5–13)	4 (3.5–4.8)	<0.001
Vascular invasion (+/−)	7/0	0/7	<0.001
Encapsulation (+/−)	3/4	7/0	0.070
Daughter nodule (+/−)	4/3	1/6	0.266
Diffentiation grade			
1	0	0	0.229
2	2	5	
3	4	2	
4	1	0	
Hepatitis			
B (−)C (−)	2	1	0.801
B (+)C (−)	4	5	
B (−)C (+)	1	1	
B (+)C (+)	0	0	
Cirrhosis (+/−)	6/1	5/2	1.000
AFP [median(range)] (ng/mL)	11.4 (2–1937)	11.0 (4.6–9198)	1.000
Survival [median(range)] (months)	4 (2.5–47)	53 (33–85)	0.002

*PVT* portal vein thrombosis, *AFP* α-fetal protein

### 2-DE analysis and protein identification

**Fig. 1:**
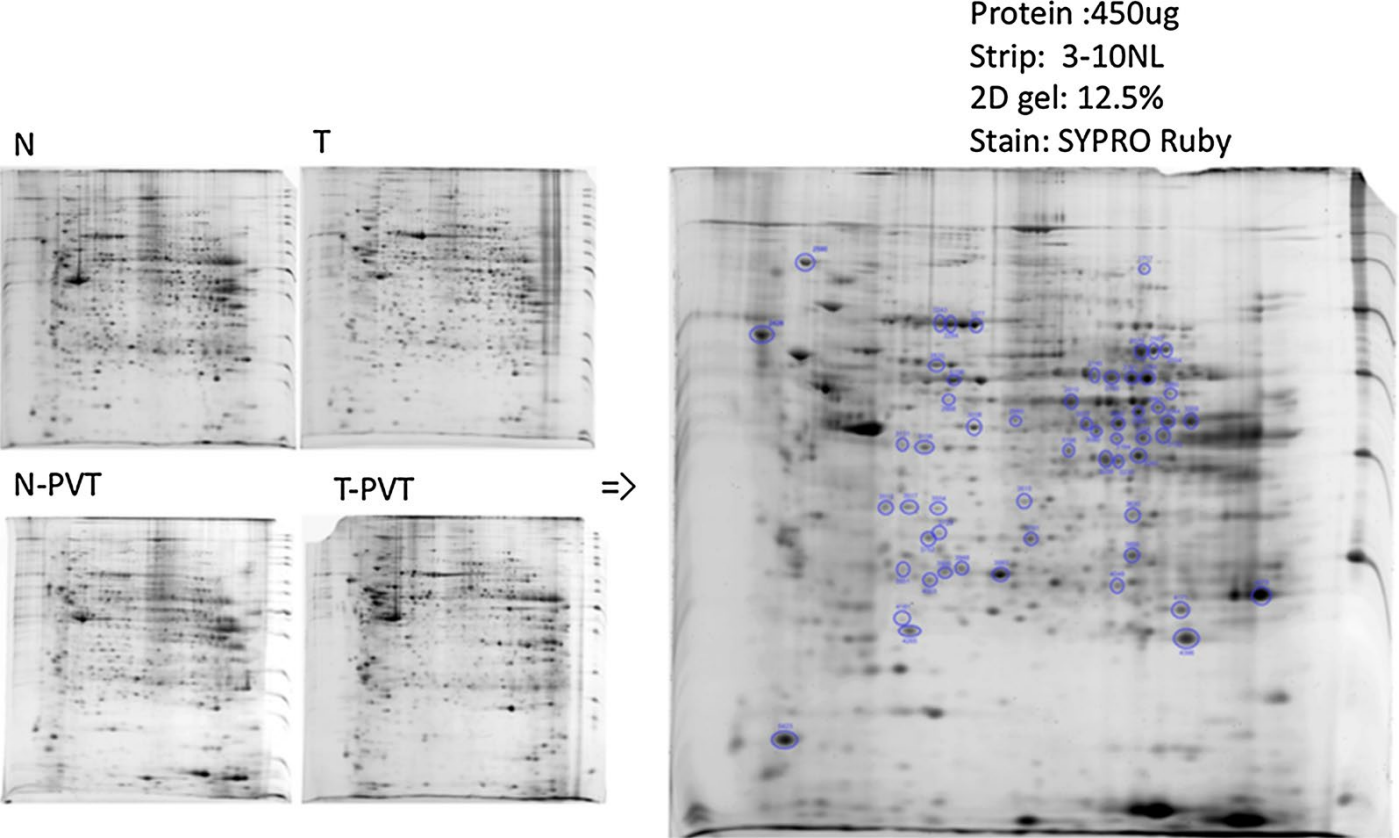
A representative of 2-D electrophoresis for HCC with or without portal vein thrombosis. *N* non-cancerous tissue from HCC without portal vein thrombosis, *T* cancer tissue from HCC without portal vein thrombosis, *N*-*PVT* non-cancerous tissue from HCC with portal vein thrombosis, *T*-*PVT* cancer tissue from HCC with portal vein thrombosis

**Fig. 2:**
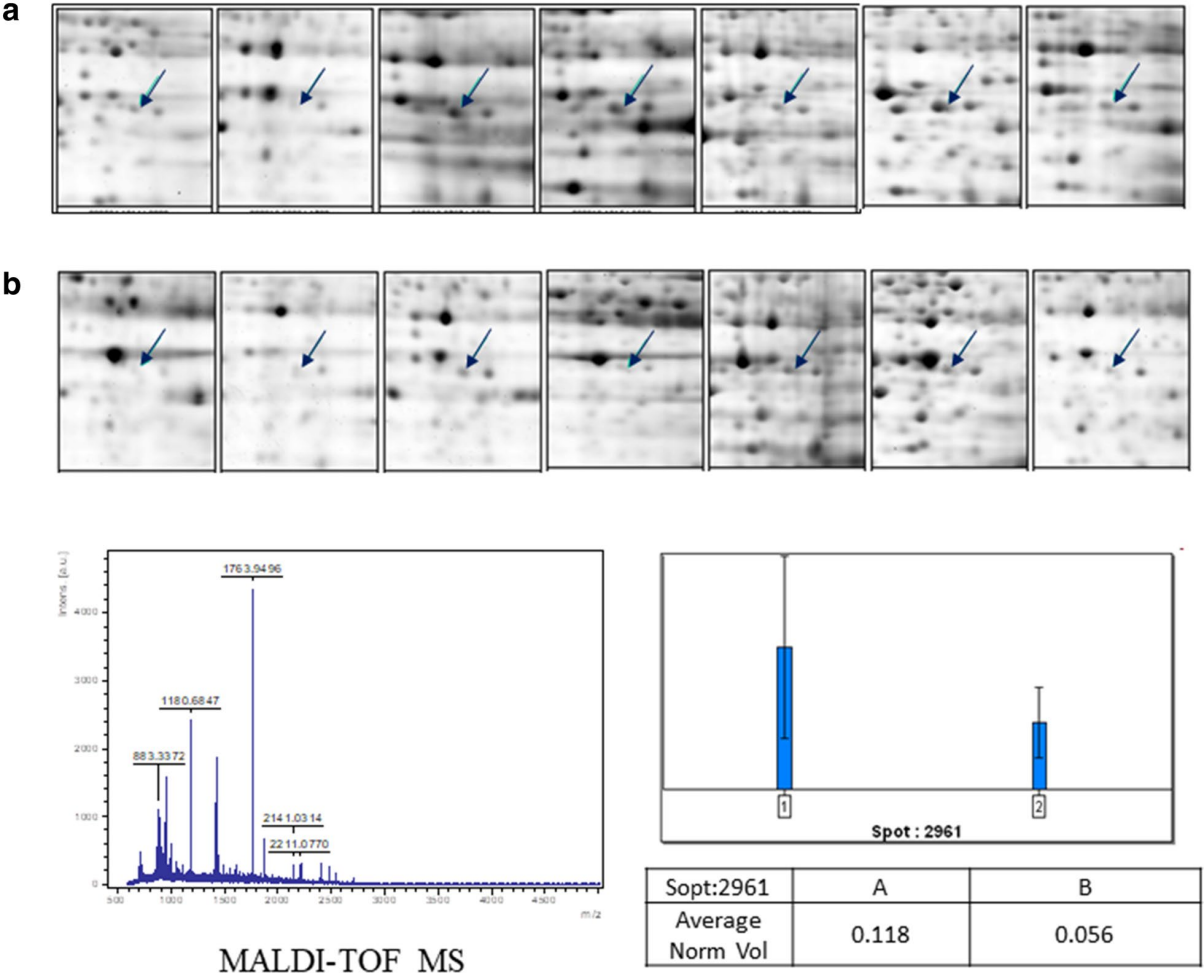
A representative of protein identification and comparison of protein expression. The 2-D electrophoresis in **a** line were obtained form cancer tissues in HCC without portal vein thrombosis and in **b** line were obtained from cancer tissues in HCC with portal vein thrombosis. The differentially expressed protein spots were identified by peptide mass matching. The protein was identified as fumarate hydratase by MALDI-TOF-MS. The gels were recorded as digitalized images and the difference of expression was calculated and compared

To find the different expression of proteins in HCC with or without PVT, proteins were extracted from 14 paired HCC tumor and adjacent noncancerous tissues to perform 2-DE separation and SYPRO Ruby staining. Representative results are shown in Fig. 1. The 14 paired specimens were analyzed on IPG strips of pH 3–10, 18 cm NL band, followed by 12.5% SDS-PAGE. The gels were scanned and images were analyzed by proxpress 2D (perkinelmer) and progenesis workstation version 2005 (nonlinear dynamics) software. The common differentially expressed protein spots were identified by MALDI-TOF-MS on the basis of peptide mass matching ( Fig. 2). In total, 45 proteins with different expression between HCC tissues with PVT or without PVT were identified. Among them, nine distinct proteins were significantly down-regulated and one was significantly up-regulated in the cancer tissues of HCC with PVT compared to adjacent non-cancerous tissues. The protein names were listed in Table 2. Among the ten proteins, the expression of fumarate hydratase (FH), carbonic anhydrase I (CA I) and dihydropteridine reductase were only down-regulated in the cancer tissues of HCC with PVT, but not down-regulated in the cancer tissues of HCC without PVT. Betaine–homocysteine *S*-methyltransferase 1 (BHMT), isovaleryl-CoA dehydrogenase (IVD), short-chain specific acyl-CoA dehydrogenase (CRAT) and arginase-1 were all down-regulated in cancer tissues of HCC without PVT and further deceased in the cancer tissues of HCC with PVT. Peroxiredoxin-4 and Endoplasmic reticulum protein ERp29 were both down-regulated in cancer tissues of HCC without PVT, but there is no difference between the cancer tissues of HCC with or without PVT. The expression of dihydropteridine reductase was not different in the cancer tissues of HCC with or without PVT, either (Table 2).

**Table 2:** The difference of protein expression in cancer and non-cancerous tissues for HCC with or without portal vein thrombosis

Spot	Protein name	Accession number	Theoretical (MW/PI)	Mascot score	Coverage (%)	Protein expression (folds)		
						N: T (*p*)	N-PVT: T-PVT (*p*)	T: T-PVT (*p*)
1717	Elongation factor 2	P13639	95,146/6.42	188	35	−1.16 (0.650)	−2.63 (0.004)	−1.43 (0.156)
2961	Fumarate hydratase	P07954	54,602/8.85	72	24	1.62 (0.256)	2.16 (0.017)	1.98 (0.088)
2966	Betaine–homocysteine *S*-methyltransferase 1	Q93088	45,426/6.7	66	30	2.64 (0.004)	4.59 (0.001)	4.30 (0.097)
3132	Isovaleryl-CoA dehydrogenase	P26440	46,803/9.3	120	35	2.13 (0.036)	3.56 (<0.001)	1.83 (0.099)
3198	Short-chain specific acyl-CoA dehydrogenase	P43155	44,611/9.1	112	35	1.56 (0.023)	3.16 (0.011)	2.38 (0.026)
3242	Arginase-1	P05089	34,713/6.87	174	52	2.67 (<0.001)	3.75 (<0.001)	2.36 (0.049)
3895	Carbonic anhydrase 1	Q13162	28,721/6.63	112	52	1.00 (0.997)	3.98 (0.001)	4.05 (0.004)
3948	Endoplasmic reticulum protein ERp29	P00915	29,032/7.5	88	40	1.89 (0.030)	3.47 (0.008)	1.89 (0.198)
4003	Peroxiredoxin-4	P30040	30,749/5.8	110	49	1.91 (0.102)	2.19 (0.002)	1.16 (1.000)
4048	Dihydropteridine reductase	P09417	26,001/7.8	70	46	1.30 (0.143)	2.29 (0.008)	1.61 (0.153)

*N* non-cancerous tissue in HCC without portal vein thrombosis, *T* cancer tissue in HCC without portal vein thrombosis, *N*-*PVT* non-cancerous tissue in HCC with portal vein thrombosis, *T*-*PVT* cancer tissue in HCC with portal vein thrombosis

#### Western blotting

**Fig. 3:**
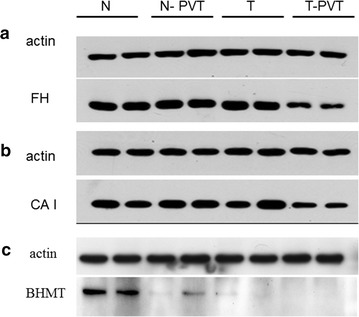
Western blots of **a** fumarate hydratase, **b** carbonic anhydrase I and **c** betaine–homocysteine *S*-methyltransferase 1. The expression of fumarate hydratase, carbonic anhydrase I, and betaine–homocysteine S-methyltransferase 1 in cancer tissues of HCC with portal vein thrombosis were down-regulated, compared to cancer tissues of HCC without portal vein thrombosis and non-cancerous tissues. This picture including 2 patients was as a representative. *N* non-cancerous tissue from HCC without portal vein thrombosis, *T* cancer tissue from HCC without portal vein thrombosis, *N*-*PVT* non-cancerous tissue from HCC with portal vein thrombosis, *T*-*PVT* cancer tissue from HCC with portal vein thrombosis

Some of differentially expressed proteins in 2-DE experiments were subjected to Western blotting analyses to confirm the differential expression. Western blotting of carbonic anhydrase I (CA I), fumarate hydratase (FH), and betaine–homocysteine *S*-methyltransferase 1 (BHMT) were performed and definitely showed they were down-regulated in the cancer tissues of HCC with PVT, compared to HCC without PVT (Fig. 3). This result was consistent with the observation in 2-DE analysis of tissue samples, showing the uniform change from non-cancerous tissue to tumor with or without PVT.

#### Immunohistochemistry

**Fig. 4:**
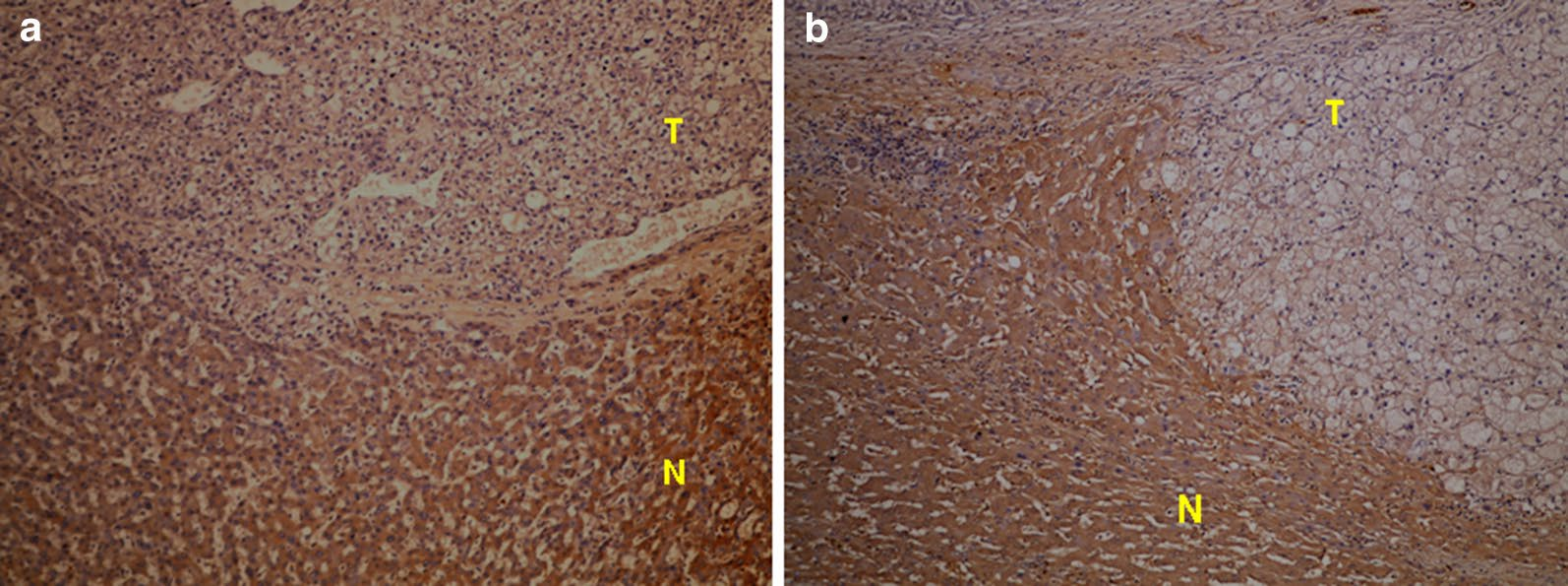
The immunohistochemical staining for carbonic anhydrase I. **a** The expression of carbonic anhydrase I in tumor (T) portion was less than non-cancer normal liver parenchyma (N) in the HCC without portal vein thrombosis. **b** The expression of carbonic anhydrase I in tumor (T) portion was less than non-cancer normal liver parenchyma (N) in the HCC with portal vein thrombosis

**Fig. 5:**
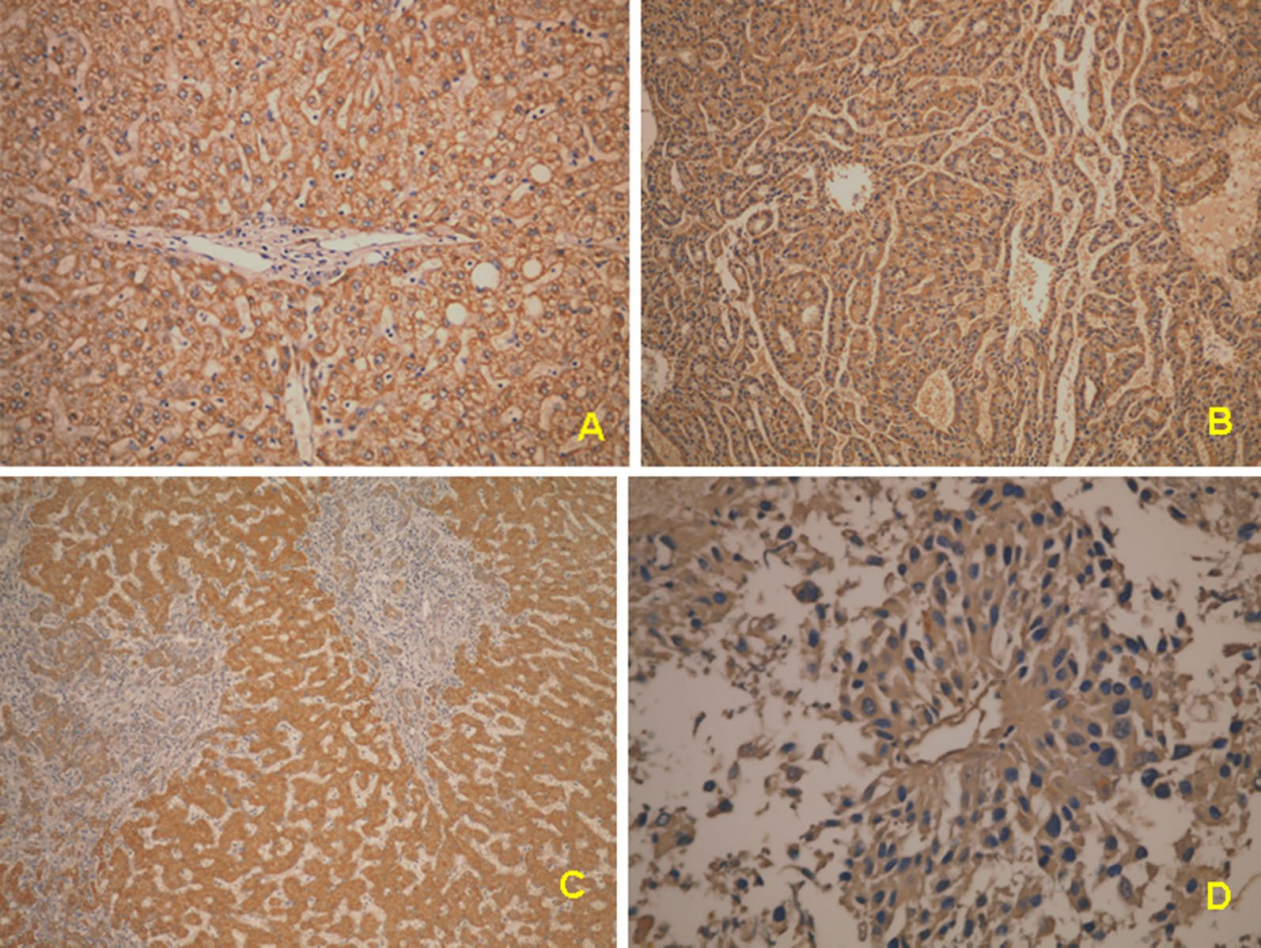
The immunohistochemistric staining for fumarate hydratase. In HCC without portal vein thrombosis, normal hepatocytes (**A**, ×100) and cancer cells (**B** ×100) both expressed fumarate hydratase. In HCC with portal vein thrombosis, normal hepatocytes (**C**, ×100) expressed fumarate hydratase. However, the cancer cells (**D**, ×200) in the veinous thrombosis expressed very low level of fumarate hydratase

To further verify the down expression of CA I and FH and distribution of CA I and FH in HCC with or without PVT, immunohistochemistry was performed to confirm the morphology of CA1 and FH expression in cancer or non-cancerous tissues. The pictures showed that normal hepatocytes expressed CA I (Fig. 4) and FH (Fig. 5) well. The expression of CA I and FH were down-regulated in HCC with PVT.

## Discussion

Portal vein thrombosis is a critical condition in the management of HCC because effective treatments are lack and the prognosis is extreme poor although sorafenib was applied to treat HCC with PVT [20]. Portal vein thrombosis was proved as an independent factor to predict short survival and even death [16]. Park et al. [17] described that the median survival time was only 2.8 months for the patients with PVT in main trunk, compared to 28.7 months for the patients without PVT. Furthermore, HCC facilitates to grow into vessels. The incidence of vascular invasion is increased when the tumor grows to a large size [13]. The most serious concern for HCC with PVT is no effective treatments: (1) liver transplantation is contraindicated; (2) liver resection can only be applied to limited number of patients with tumor limited in either lobe of the liver; and (3) non-surgical treatments including hepatic artery infusion of chemo-agents (HAIC), transarterial chemembolization, transarterial radioembolization with yttrium-90, or combination of HAIC and radiotherapy extended survival only for a few months [21–24]. Therefore, to understand the possible reasons/mechanisms of HCC invading vessels is essential for effective treatment of HCC.

Protein expression reflects and contributes the function of cells. In this study, the aims focused on why HCC invaded portal vein and form thrombosis. Roughly, 45 proteins with different expression on HCC with PVT or HCC without microvascular invasion were identified. But, when we focused on the protein expression with fold-difference to adjacent noncancerous tissue or cancer tissue without PVT forming, FH, CA I, BHMT, IVD, CRAT, and arginase-1 were the six proteins which expressed deficiently in cancer cells with PVT. All these proteins belonged to the enzymes of cellular metabolism and acid–base balance. Obviously, the microenvironment in tumors with PVT was altered. The cellular metabolisms of the cancer cells in HCC with PVT were altered, too.

According to results, FH and CA I were the two proteins that decreased in cancer tissues with PVT, but not in the cancer tissues without PVT. Fumarate hydratase is an important enzyme working in Kreb’s tricarboxylic acid cycle. Fumarate hydratase catalyses the hydration of fumarate to form malate. Deficiency of FH would result in accumulation of fumarate in mitochondria, leak into cytosol and inhibit prolyl hydroxylase which suppresses the expression of hypoxia-inducible factor-1 (HIF-1) [25–27]. In this study, the FH expression in cancer cells with PVT was more than 2-fold decreased. Decreased FH would impair oxidative phosphorylation in cancer cells and force these cancer cells to depend on glycolysis for energy production under aerobic condition. This pseudohypoxia response will further induce hypoxia-inducible transcription factor [28]. Ashrafian et al. [29] isolated primary mouse embryonic fibroblasts from FH 1-deficiency mice and confirmed that these fibroblasts would depend on glycolysis for energy production, elevated rate of lactate efflux and up-regulate HIF-1 and HIF target genes. Recent study also showed that accumulation of metabolites of Kreb’s tricarboxylic acid cycle in tumor cells caused epithelial-to-mesenchymal transition and renal cancer cells became aggressive when oncometabolite fumarate accumulated due to FH deficiency [30]. The down-regulation of FH might let HCC cells become aggressive and easy to grow into vessels [27, 31].

Carbonic anhydrases is a family of zinc metalloenzymes that catalyze the interconversion between carbon dioxide and bicarbonate ion to acid–base balance in cellular environment [32]. Up- or down-regulation of carbonic anhydrases in cancer cells or other stromal cells will change the acid-based balance in the microenvironment of tumors. Carbonic anhydrase I located in cytosol catalyzes the interconversion between bicarbonate ion and carbon dioxide to facilitate carbon dioxide diffusing cross cell membrane. During anaerobic metabolism, hydrogen ion produced within the cells needs cytosolic CA to catalyze the conversion reaction from hydrogen ion and bicarbonate toward carbon dioxide [32]. Decreased expression of CA I would result in cellular acidic PH which promote tumor motility and contribute to tumor growth and metastasis [33]. It has been reported that reduced expression of CA I and II in poor differentiated HCC [34]. In this study, CA I expression in the tumor with PVT was much lower than in the tumor without PVT. This result might explain that tumors with PVT became oxygentactic and aggressive to invade vessels.

In this study, we also found that BHMT, IVD, CRAT and arginase-1 were all down-regulated in cancer tissues of HCC without PVT and further deceased in the cancer tissues of HCC with PVT. BHMT is a cytosolic enzyme that catalyses the conversion of homocysteine to methionine. Teng et al. showed BHMT was important in one-carbon metabolism and lack of BHMT resulted in fatty liver and HCC in an animal model [35]. Yan et all also showed that deficiency of BHMT leads to accumulation of homocysteine which impairs endothelial function by compromising VEGF/Akt endothelial nitric oxide synthetase signaling and vascular complications [36]. Isovaryl-CoA dehydrogenase is a mitochondrial matrix enzyme that catalyzes the third step in leucine metabolism. Clinically, reduced activity of IVD is a recessive autosomal disorder and cause isovaleric acidosis [37]. Clinical symptom is presented as severe metabolic acidosis. Deficiency of IVD in HCC cells might cause accelerate acidosis in the microenvironment of tumor which might exaggerate cancer to invade vessels and resistant to chemotherapy or radiotherapy. In this study, CRAT was also down-regulated in the cancer cells with PVT. Clinically, deficiency of CRAT is an autosomal recessive inborn error of mitochondrial fatty acid oxidation [38]. Hypoglycemia is one of the clinical presentations. Imaginably, decreased level of glucose in the tumor microenvironment might force cancer cells to invade vessel to get energy source.

The dysregulation of metastatic enzymes in HCC with PVT found in this study were very similar to those in renal cell carcinoma (RCC). Renal cell carcinoma is one of the most easy-to-recur tumors in United States. Clinically, RCC also has the facilitation to grow into vessels and form a tumor thrombus in renal veins or inferior vena cava. Dorai et al. [39] reviewed the molecular evidence in different types of RCC and described a common pathway of deficiency of FH and dysregulation of CAs. Pseudohypoxia with glycolytic metabolism combined with microenvironment acidosis might induce hypoxia-inducing factor and vascular endothelial growth factor. Molecular targeting therapy with sunitinib or sorafenib has been proved to be effective to treat metastatic RCC clinically [40, 41]. Sorafanib is also used to treat advanced HCC already [42, 43]. Based on molecular finding in this study, molecular targeting therapy may be effective to treat HCC with vascular invasion by blocking the downstream pathway of metabolic enzyme dysregulation.

## Conclusion

HCC with PVT is an aggressive cancer with extremely poor prognosis. Compared to the protein expression in HCC without PVT, HCC with PVT showed decreased expression of several metabolic enzymes and carbonic anhydrases: FH, CA I, BHMT, IVD, CRAT, and arginase-1. This deficiency of metabolic enzymes and cytosol CA may facilitate HCC to invade portal vein for nutrient and oxygen supply and form a thrombus. Targeting these metabolic enzymes and cytosol CA may help the treatments for HCC with PVT.

## Abbreviations

HCC: hepatocellular carcinoma
PVT: portal vein thrombosis
CA I: carbonic anhydrase
FH: fumarate hydratase
BHMT: betaine–homocysteine *S*-methyltransferase 1
IVD: isovaleryl-CoA dehydrogenase
CRAT: short-chain specific acyl-CoA dehydrogenase
